# Supplementary material for: Recurrent humid phases in Arabia over the past 8 million years
Source: Nature. 2025 Apr 9;640(8060):954–61. doi: 10.1038/s41586-025-08859-6 (PMC12018461; doi:10.1038/s41586-025-08859-6)
Supplement: Supplementary file 1 — This file provides a key to the tabs in Supplementary Data 1. [file 41586_2025_8859_MOESM1_ESM.pdf]

nature portfolio

https://doi.org/10.1038/s41586-025-08859-6

Supplementary information

Recurrent humid phases in Arabia over the past 8 million years

In the format provided by the authors and unedited

Guide to tabs in Supplementary Information Excel file

Tab 1. U-Th solution chemistry derived ages and uncertainties.

Tab 2. U-Th laser ablation derived ages and uncertainties.

Tab 3. Instrumental setting for U-Th laser ablation.

Tab 4. Instrumental cup configuration for U-Th laser ablation.

Tab 5. U-Pb calculated ages and uncertainties.

Tab 6. U-Pb age clusters discussed in the main text of the manuscript.

Tab 7. The measured $^{234}U/^{238}U$ activity ratio and the calculated initial $^{234}U/^{238}U$ ratio.

Tab 8. U-Pb measured data part 1.

Tab 9. U-Pb measured data part 2.

Tab 10. U-Pb reference material part 1.

Tab 11. U-Pb reference material part 2.

Tab 12. Meta Data for the U-Pb analyses.

Tab 13. Summary of all stable isotope carbonate data for all speleothems analysed.

Tab 14-34. Stable isotope carbonate data for all speleothems analysed.

Tab 35. Fluid inclusion stable isotope data of all speleothems analysed.
